# High-Density Linkage Map and QTLs for Growth in Snapper (*Chrysophrys auratus*)

**DOI:** 10.1534/g3.118.200905

**Published:** 2019-02-25

**Authors:** David T. Ashton, Peter A. Ritchie, Maren Wellenreuther

**Affiliations:** * The New Zealand Institute for Plant & Food Research Limited, Nelson, New Zealand; † School of Biological Sciences, Victoria University of Wellington, Wellington, New Zealand

**Keywords:** linkage map, genome, fish, QTLs, growth, genotyping-by-sequencing

## Abstract

Characterizing the genetic variation underlying phenotypic traits is a central objective in biological research. This research has been hampered in the past by the limited genomic resources available for most non-model species. However, recent advances in sequencing technologies and related genotyping methods are rapidly changing this. Here we report the use of genome-wide SNP data from the ecologically and commercially important marine fish species *Chrysophrys auratus* (snapper) to 1) construct the first linkage map for this species, 2) scan for growth QTL, and 3) search for putative candidate genes in the surrounding QTL regions. The newly constructed linkage map contained ∼11K SNP markers and is one of the densest maps to date in the fish family Sparidae. Comparisons with genome scaffolds of the recently assembled snapper genome indicated that marker placement was mostly consistent between the scaffolds and linkage map (R = 0.7), but that at fine scales (< 5 cM) some precision limitations occurred. Of the 24 linkage groups, which likely reflect the 24 chromosomes of this species, three were found to contain QTL with genome-wide significance for growth-related traits. A scan of 13 candidate growth genes located the *growth hormone, myogenin, and parvalbumin* genes within 5.3, 9.6, and 25.0 cM of these QTL, respectively. The linkage map and QTL found in this study will advance the investigation of genome structure and aquaculture breeding efforts in this and related species.

Characterizing the genetic variation that affects phenotypic traits is a central goal in biology. Understanding this variation can inform selective breeding programs (Dekkers 2012), be used to predict disease risk in medicine (Lehner 2013), and help researchers to understand evolution in natural populations (Savolainen *et al.* 2013). While genetic research has typically been pioneered in laboratory model species, the development of affordable high-throughput genomic methods (*e.g.*, next generation sequencing) is now allowing this research to be extended to a wide range of non-model species (Hilario 2015; Braasch *et al.* 2015).

Locating and characterizing quantitative trait loci (QTL) is one commonly used approach to investigate how genetic variation influences a specific phenotype (Pértille *et al.* 2017; *e.g.*, Barria *et al.* 2017; Chen *et al.* 2017). QTL mapping methods locate molecular marker loci influencing a phenotypic trait based on a significant correlation between the allelic variation of that loci and variation of the trait (Lynch and Walsh 1998). New QTL can be informative as standalone observations, or used to identify candidate genes in the surrounding genome, which may be influencing the trait (*e.g.*, Bettembourg *et al.* 2017). It should be noted, however, that QTL studies and associated candidate gene investigations should be seen as a first step to gain insights into the genotype-phenotype map, and that additional studies are needed to verify that candidate genes are indeed causal in affecting the trait in question. Such genotype-phenotype datasets can also be used to develop multi-marker models (based on multiple QTL), which explain variation of one or more phenotypic traits (Cros *et al.* 2017).

Having a road-map of the genome (*e.g.*, a linkage map or high quality genome assembly) is an important prerequisite for QTL mapping, as it allows the relative positioning of different marker loci. High-quality genome assemblies are most effective because they allow genetic markers to be positioned at a base-pair level, while also providing sequence information for the surrounding area. However, most non-model species do not currently have chromosome-level genome assemblies and instead rely on linkage maps to ascertain the relative position of markers in the genome (Braasch *et al.* 2015). Although they are often less precise than high quality genome assemblies that have been built with multiple datasets, linkage maps can serve the dual purpose of bridging the resource gap before a genome is developed and providing useful information to improve the arrangement of scaffolds during the genome assembly process (Fierst 2015). Indeed, data sets that are developed for constructing a linkage map can also be used in QTL mapping if phenotypic data are available.

Teleost fish show significant potential to benefit from more affordable and high-throughput genomic technologies for a number of reasons. Teleosts are the largest group of vertebrates, with over 26,000 species (Miller and Harley 2006), and most species have a limited geographic range. Furthermore, several species are of commercial significance because they either have large natural stocks that are harvested by the fishing industry, or they are used in selective breeding programs in aquaculture. The restricted geographic range combined with this high diversity of species that are commercially exploited in wild fisheries and aquaculture means that scientific efforts are spread thinly overall, with few to no genomic resources available for many species.

One of the most important traits in farmed species is growth performance, as it directly affects the efficiency of production systems. Selective breeding programs have been successful to enhance growth gains both terrestrial (VanRaden *et al.* 2009) and aquatic animals (*e.g.*, Murata *et al.* 1996). In most animal species, growth is a complex trait that is influenced by a network of genes (De-Santis and Jerry 2007) and multiple environmental factors, such as seasonal variation in temperature, food availability, and competition (Handeland *et al.* 2008). Moreover, growth is also commonly correlated with variation in other life-history traits, such as gonad maturation processes and reproductive timing (Bhatta *et al.* 2012; Park *et al.* 2016). Despite the numerous factors influencing growth, most quantitative genetics studies that investigate growth report moderate to high heritabilities (*e.g.*, 0.1 - 0.5) in a wide range of taxa (Wang 2009; Tsai *et al.* 2015; Ye *et al.* 2017). In fish, a number of genes associated with growth have been identified (reviewed in De-Santis and Jerry 2007), including *growth hormone*, insulin-like growth factors, as well as a range of myogenic growth regulators.

Here we focus on the marine teleost *Chrysophrys auratus* (family: Sparidae), commonly referred to as the Australasian snapper (henceforth referred to as “snapper”). Snapper supports a valuable recreational and commercial inshore fishery around the northern parts of New Zealand, southern Australia, and some of the Pacific Islands (Parsons *et al.* 2014), and is a strong candidate for development into an aquaculture species in both New Zealand and Australia. Closely related sparid species are already used for several aquaculture breeding programs around the world, for example, the sister species of snapper *Pagrus major* accounts for 10% of the total value of aquaculture in Japan. As part of our long-term research program to develop snapper into an aquaculture ready species, we here seek to identify genetic variation underlying growth differences among individuals in the breeding program to aid the selection of high-quality broodstock. In particular, our specific objectives were to use genome-wide SNP data from our pedigreed population to 1) construct a high density linkage map, 2) conduct QTL mapping for three measures of growth (peduncle length, fork length, and weight), and 3) investigate the position of 13 candidate growth genes and their relative position to growth QTL.

## Materials and Methods

### Study population

A snapper breeding program was started at The New Zealand Institute for Plant & Food Research Limited in 2016 and includes a population with three generations held at the Nelson Research Centre in New Zealand. Data from the two most recent generations (F_1_ = 70 individuals, F_2_ = 577 individuals) were investigated in this study. Uncontrolled mass spawning of the F_1_ generation in a single tank was used to produce the offspring F_2_ generation. This resulted in a complex pedigree, meaning that we obtained a combination of full-siblings, half-siblings, and unrelated individuals in the F_2_ generation (Supplementary Table 1). The F_2_ offspring were held in a single tank until they were approximately one year old and then split evenly among four tanks with comparable feeding, light, water flow, aeration, tank design. All research carried out in this study was reviewed and approved by the animal ethics committee of Victoria University of Wellington in New Zealand (Application number 2014R19).

### Phenotyping

Three measures of growth were used in the current study namely fork length, peduncle length, and weight. Fork length was measured as the distance from the nose to the fork in the tail. Peduncle length was measures as the distance from the nose to the narrowest cross-section across the tail. Measurements were made when the fish were a little over one year old (between 436-487 days) and again when they were approximately three years old (1045-1131 days). Length measurements were made by collecting images of each individual and then making measurements from those images. A ruler was included in each image to provide a scale. The number of individuals measured differed between year one and year three as a result of natural mortality during the study.

### Genotyping

Samples of fin tissue were collected for all fish and DNA was extracted from these samples using a modified salt extraction protocol (Aljanabi and Martinez 1997). Quantification of DNA was carried out using Hoescht 33258 fluorescent dye. Fragmentation of the extracted DNA was checked by gel electrophoresis. Samples with moderate (∼25%) amounts of fragments below 10 kbp were re-extracted and if needed fresh samples were collected.

Only high quality genomic DNA was used for the preparation of Genotyping By Sequencing (GBS) libraries based on the protocol described by Elshire *et al.* (2011). For each library, one microgram of DNA was double digested with the restriction enzymes *Pst*I and *Msp*I. The adaptor ligation step was done after digestion, without allowing the DNA/adaptor mixture to dry out. The barcoded adaptors were designed by Deena Bioinformatics and bound to the *Pst*I cut sites. Adaptors were subsequently annealed according to the method of Ko *et al.* (2003). The high fidelity enzyme AccuPrime Taq DNA polymerase High Fidelity (Life Technologies) was used for amplifications. Each library was amplified separately and its quality assessed by capillary electrophoresis prior to sequencing (Fragment Analyzer, Advanced Analytical). All GBS libraries were prepared in parallel in plates. Duplicate or triplicate samples were prepared for each of the parent and grandparents and single samples for each of the offspring (except for three individuals that had poor DNA quality, for which duplicate samples were prepared). Each plate (containing 96 individual libraries) was pooled, then cleaned up, quantified and sent to the Australian Genome Research Facility (AGRF) in Melbourne, Australia, for sequencing. Each pool was sequenced on a single lane with the Illumina HiSeq 2500 platform in single end (SE) mode, with a read length of 100 bases. In total, eight pools of libraries were sequenced in eight lanes for this project.

FastQC was used to conduct an initial check of the sequencing data quality. Sequences were then de-multiplexed and cleaned. Adapters and primers were removed and the sequencing data were cleaned using Fastq-mcf in the ea-utils package (Aronesty 2011). Genotyping was carried out on the cleaned datasets using STACKs v1.40 (Catchen *et al.* 2013). The samples were first demultiplexed from the eight sequencing libraries using the process_radtags module command. Then sequencing reads for the duplicate or triplicate samples were concatenated into a single file, after which the reads were trimmed using Fastq-mcf with a minimum sequence length of 50, and a quality threshold causing base removal of 33. Bowtie v1.0 was used to align the GBS data to the genome assembly (with the ‘ref_map.pl’ option), allowing for 3 mismatches and 10, reported alignments. The pstacks module was then run, only including data that had a minimum coverage of 8x, followed by cstacks and sstacks using the pre-set parameters for the latter two modules. The population module was then used to output the data to a Genepop file while further filtering the data by applying a minor allele frequency/MAF of 0.05 and allowing only 0.25 of missing data. These SNP filtering steps were used to minimize missing data, exclude putative sequencing errors and to have sufficient power to call heterozygotes, while keeping a substantial number of informative SNPs. After these filtering steps a total of 20,311 SNPs were retained for subsequent analyses.

### Linkage map construction

The parents for each F_2_ individual in the dataset were identified using CERVUS v3.0.7 (Kalinowski *et al.* 2007) and a subset of SNPs (n = 2174) that were present in >98% of individuals. All parental pairs were selected with a 95% confidence level based on the built in permutation procedure. A linkage map was constructed based on the SNP and pedigree data in LEPMAP v2.0 (Rastas *et al.* 2016). Data from the largest 14 F_2_ families (full and half-sibling families) were used, and included a total of 269 offspring and 14 parents, and reduced the total number of available SNPs for this analyses to 20,311 SNPs. Markers were separated into chromosomes with the SeparateChromosomes module (logarithm of odds (LOD) limit = 14, minimum markers per linkage group = 50). The marker order was then generated with the OrderMarkers module. Markers near the start and end of each linkage group (start and end 10% based on centimorgan (cM) distance) were removed if they were more than 3 cM from the next closest marker. The accuracy of the final linkage map was investigated by comparing the linkage map position (cM) with the position of markers on available genome scaffolds (base-pairs) from the genome assembly (number of scaffolds 5998). The scaffold and base-pair position for each marker in the linkage map was retrieved from the STACKS v1.40 output files. Using this information the correlation between linkage map (cM) and scaffold (base-pair) position was tested for all scaffolds that contained >50 SNPs. The mean and 95% confidence interval of the correlation residuals was then calculated. Whether scaffolds were placed uniquely on one of the 24 expected linkage groups was also investigated as well as the number and total base-pairs of scaffolds able to be positioned on the linkage group. The extent of linkage disequilibrium across the linkage groups was reviewed by calculating the pairwise linkage disequilibrium results for each set of markers using PLINK v1.9 (Purcell *et al.* 2007) and then visualizing the mean value at different distances across the linkage groups in R statistical environment v3.2.3 (R Core Team 2013). This was done for all individuals in the F_2_ generation and separately for individuals in the largest full sibling F_2_ family (n = 48). The sex-specific recombination rate was calculated by comparing the linkage map distance (cM) and genome scaffold distance (bp) between individual marker pairs for males and females.

### QTL identification

Quantitative trait loci identification was carried out using the general model implemented in QTDT v2.6.1 (Abecasis *et al.* 2000) and the half-sibling regression method implemented in GRIDQTL v3.3.0 (Knott *et al.* 1996; Seaton *et al.* 2006). Both methods utilize parents as controls for population stratification and can use multiple offspring per family from a complex pedigree (Knott *et al.* 1996; Abecasis *et al.* 2000). Genotype data from the F_1_ and F_2_ generations and phenotyping data from the F_2_ generation were used. QTDT used 10,716 markers which had been placed on the linkage map. GRIDQTL used a subset of markers (n = 1007), which were filtered randomly to a minimum spacing of 1 cM. Before running the analysis, the genotype data were filtered for Mendelian errors by dropping loci for any individual that contained alleles not observed in either of the two parents. The phenotype measurements used for the analysis were standardized by tank and date collected to correct for temporal and tank effects. The QTL scan results from QTDT were visualized using the ggplot2 library v3.1.0 in the R statistical environment v3.2.3 (R Core Team 2013). The genome-wide 95% confidence limits were calculated for QTDT using a Bonferroni correction (*i.e.*, 0.05 / 10,716 markers) and using the built in permutation procedure in GRIDQTL with 1000 permutations.

### Candidate genes and their location

The position of 13 candidate growth genes for fish (De-Santis and Jerry 2007) and growth QTL identified in this study were compared using the genome assembly. To do this, the sequence for each candidate gene was located on the NCBIS nucleotide database from the closest related teleost species - either the DNA or mRNA sequence. DNA sequences were mapped to the genome scaffolds by selecting the largest exon for the target gene and aligning with the “Map to Reference” alignment using the “Geneious mapper” in Geneious v10.0.9 (Kearse *et al.* 2012); alignment sensitivity was set to “High Sensitivity / Medium” with default settings. For mRNA sequences the sequences were aligned with the “Map to Reference” alignment using the “RNA Seq” mapper in Geneious; alignment sensitivity was set to “High Sensitivity / Medium” with the maximum gap size increased to 1000 bp. For each alignment the percentage of matching base pairs was reported for the largest exon. The linkage group and cM position of the scaffold containing specific candidate genes was then located using the STACKs output files and the newly constructed linkage map. Candidate gene locations were then compared with the position of putative growth QTL peaks (GRIDQTL) or closest genome-wide significant marker (QTDT).

### Data availability

All data used in this study including the genome assembly, GBS sequencing libraries, phenotype data and supplemental material are located in an open data repository which can be accessed via https://www.genomics-aotearoa.org.nz/data.

## Results

### Phenotyping

**Figure 1:**
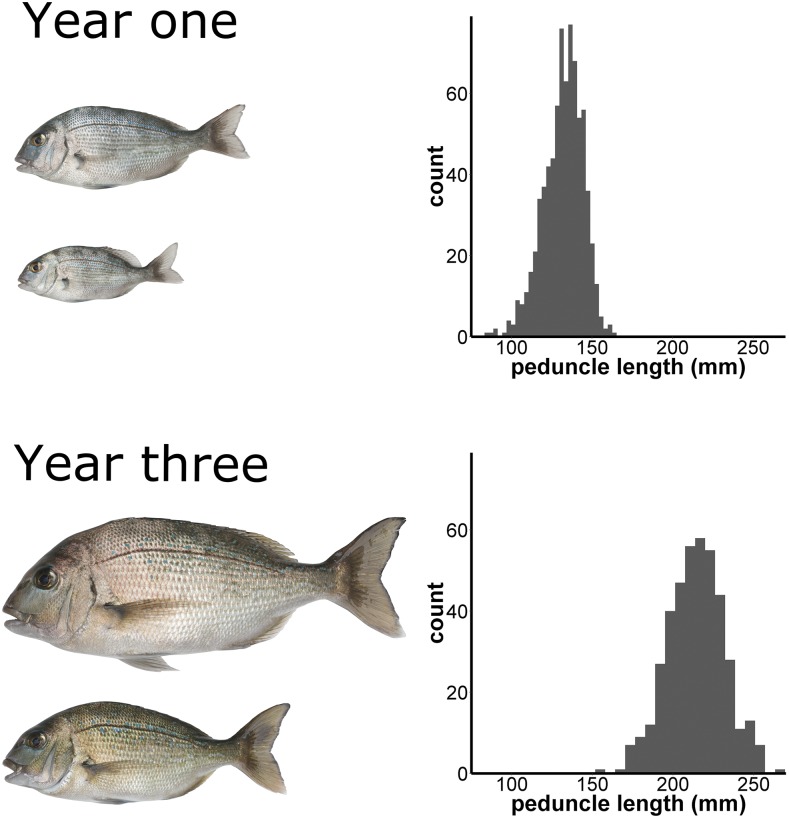
Peduncle length measurements at year one (n = 568) and year three (n = 314). Images on the left side of the diagram are to scale relative to each other and show the smallest and largest individual fish from a tank of F_2_ individuals at year one and year three, respectively.

Peduncle length, fork length, and weight were recorded when individuals where 436-487 days old (year one) and 1045-1131 days old (year three). The distribution and relative sizes of fish in year one and year three are illustrated in Figure 1. In the first set of measurements the mean and standard deviation for fork length, peduncle length, and weight were 160.1 ± 15.0 mm, 132.1 ± 12.3 mm, and 75.5 ± 20.3 g, respectively (Table 1). In the second set of measurements the same measures were 257.8 ± 20.2, 214.5 ± 17.0, and 361.9 ± 82.3, respectively (Table 1). The three measures for growth were all found to be strongly positively correlated (Pearson’s R > 0.93, Ashton *et al.* 2019). Strong positive correlation was also observed between year one and year three for each measure (Pearson’s R = 0.71 – 0.73; Supplementary figure 1).

**Table 1:** Peduncle length, fork length, and weight at year one and year three including number or measurements (n), mean, and standard deviation (stdev)

	Year one			Year three		
	n	mean	stdev	n	mean	stdev
**Peduncle length (mm)**	568	132.1	12.3	314	214.5	17.0
**Fork length (mm)**	568	160.1	15.0	314	257.8	20.2
**Weight (g)**	530	75.5	20.3	247	361.9	82.3

### Genotyping by sequencing

A total of 1.6 billion reads were produced for all eight pooled GBS libraries with approximately 2, 4 or 6 million reads for each single, duplicate, or triplicate individual library respectively. Using the STACKs pipeline a total of 20,311 SNPs were found after filtering for >7x coverage, present in 75% of the individuals in the population, and a minor allele frequency (MAF) of 0.05. The average coverage per SNP was 15.6x in the offspring (F_1_) and 23.9x in the parents (F_2_).

### Pedigree structure and linkage map

Parents were identified for 93% of the individuals in the F_1_ and F_2_ generations. The remaining 7% with missing parents were mainly located in the F_1_ generation, and were the result missing F_0_ wild-caught individuals that were not available for sampling at the time of the study. A mixture of 127 full sibling and half sibling families were present in the F_2_ generation.

**Figure 2:**
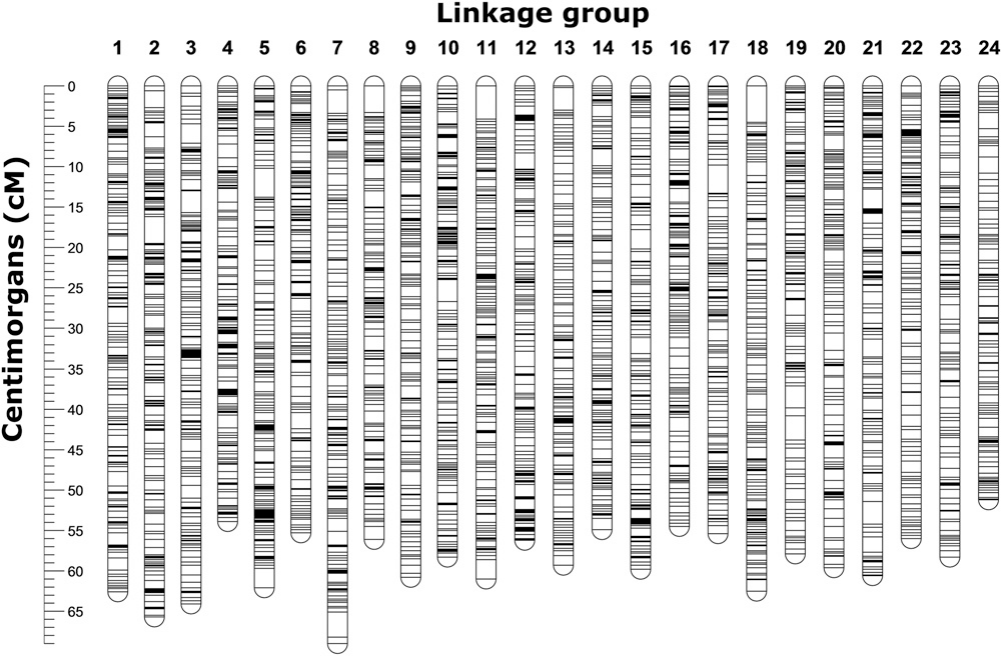
Visualization of the linkage map including a total of 10,716 SNPs placed along the map in 24 linkage groups. The 24 linkage groups are equivalent size and number to represent the expected 24 *Chyrsophrys auratus* chromosomes.

**Figure 3:**
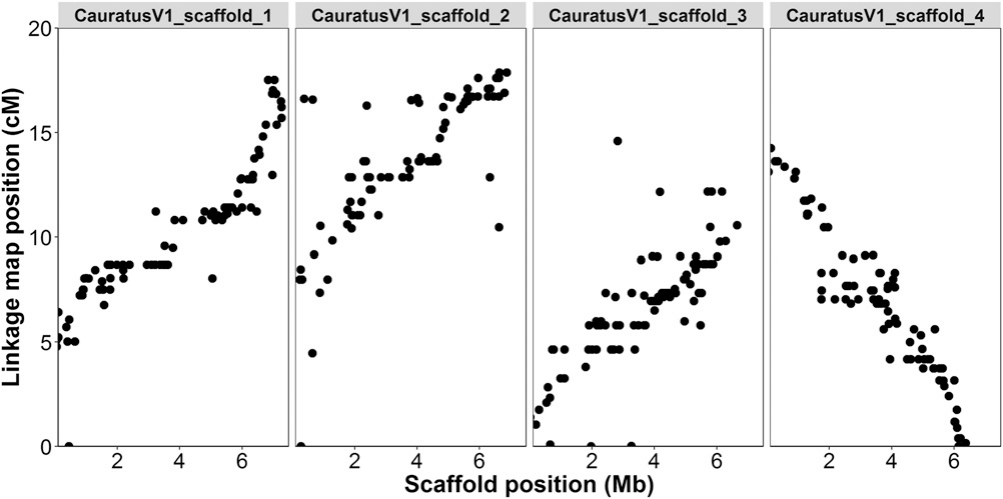
Comparison of linkage group (cM) and genome scaffold (Mb) position for loci placed on the four largest scaffolds available from the genome. The position was strongly correlated between the two approaches (scaffold_1 = 0.91, scaffold_2 = 0.53, scaffold_3 = 0.78, scaffold_4 = -0.95), but there is also noise around the precise placement on the linkage map.

**Figure 4:**
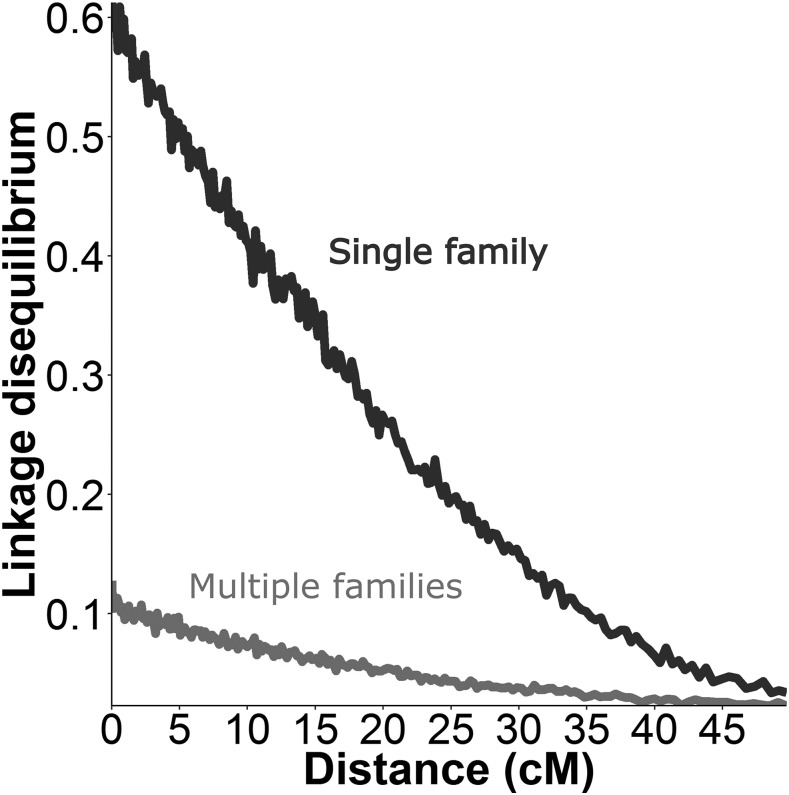
Linkage (R_2_ correlation) between each pair of markers in the dataset plotted against the distance (cM) between those markers on the linkage map. This statistic was calculated for the entire QTL mapping dataset (multiple families, families = 137, n = 539) and for the data from the single largest family in the dataset (n = 48). The results show a consistent decay of linkage across the length of the linkage maps and > 5x higher linkage in the largest family than the entire dataset.

A total of 10,716 SNPs were positioned on the linkage map (Figure 2). The total length of the sex-averaged linkage map was 1,363.0 cM with an average marker spacing of 0.129 cM. The lengths of the male and female maps were 1401.5 cM and 1359.0 cM, respectively. The female and male recombination rates were 3.28 cM/Mb and 1.93 cM/Mb based on comparison with available scaffolds from the snapper genome assembly. Moderate correlation (R = 0.74 ± 0.20 for 1723 markers on 26 scaffolds) was found across all scaffolds with >50 markers. The 95% confidence interval of the correlation residuals ranged from -5.7 to 3.2 centimorgans with a mean of -1.25; indicating that 95% of markers were placed within ∼4.5 cM of their base-pair location. Visualization of the four largest scaffolds showed a clear relationship between the ordering of markers (correlation = 0.91, 0.53, 0.78, and -0.95), but that some noise was apparent around the exact placement (Figure 3). When aligning the genome scaffolds to the linkage map, individual scaffolds were placed exclusively onto one of the 24 linkage groups. Scaffolds which included markers from the linkage map contained a total of 701 Mb or ∼95% of the total base-pairs in the snapper genome. Investigation of the degrees of linkage and linkage disequilibrium within the dataset showed a clear pattern of linkage decay over the length of the linkage groups (Figure 4). When looking at a single F_2_ family there was a high degree of linkage. However, when looking at the whole F_2_ generation the decay of linkage and linkage disequilibrium is much greater, with minimal linkage observed even over small distances.

### QTL mapping

**Figure 5:**
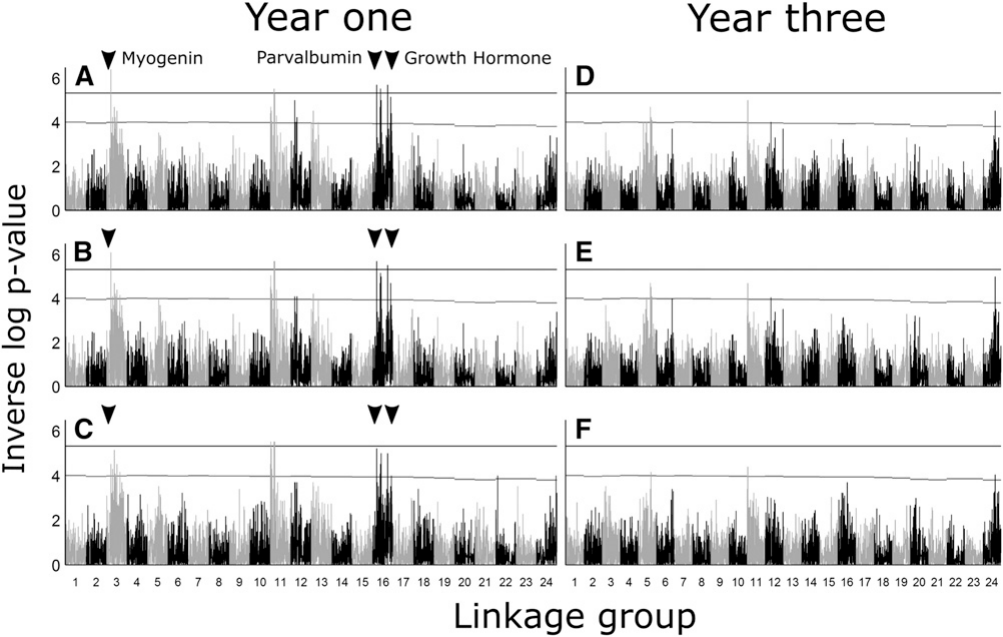
QTL scans at year one for fork length (A and D), peduncle length (B and E), and weight (C and F) using QTDT and GridQTL. The black horizontal line on each graph indicates the 95% genome-wide significance level. Genome-wide significant QTL were found using both software on linkage groups 3 and 16. A third genome-wide significant QTL was found on linkage group 11 using QTDT, but not GridQTL. QTL were shared between all traits within each software, except for weight in QTDT which had no genome-wide significant QTL.

Multiple QTL were found for all three growth traits in year one (Figure 5, Table 2, and Supplementary table 2). Genome-wide significant QTL were located on linkage groups 3, 11, 16 for fork length and peduncle length in QTDT and linkage groups 3, and 16 for all growth traits in GRIDQTL. The length trait QTL on linkage groups 3, 11, and 16 from QTDT were significant at a chromosome wide level for weight. The genome-wide significant markers for QTDT in year one had moderate effect sizes ranging from a minimum R^2^ of 0.04 to a maximum of 0.05 for markers in QTDT (Table 2). No genome-wide significant QTL were found for growth traits in year three.

**Table 2:** Putative QTL markers (QTDT) that were significant for at least one trait at a genome wide significance level of 5.33. Effect size (R^2^) was estimated in QTDT as the difference between the R squared values of the total model and the genotype model. Loci are reported including their linkage group number (LG) and position (cM). Genome wide (**) and linkage group wide (*) significance are indicated with asterix

			Fork length		Peduncle length		Weight	
LG	cM	loci	-log10(p)	R^2^	-log10(p)	R^2^	-log10(p)	R^2^
3	20.9	40730_33	6.4**	0.04	5.7**	0.04	4.3*	0.03
11	10	99093_35	5.7**	0.05	5.7**	0.04	5.22*	0.04
16	47.9	62074_47	5.7**	0.05	5.5**	0.04	4.52*	0.03
16	40.2	39092_27	5.52**	0.05	5.22*	0.05	4.52*	0.05

### Candidate genes

The base-pair position on the genome scaffolds were found for all 13 candidate genes including *growth hormone, growth hormone receptor, growth hormone receptor type 1, growth hormone receptor type 2, insulin like growth factor 1, insulin like growth factor 2, myogenic factor 1, myogenic factor 2, myogenic regulatory factor 4, myogenic regulatory factor 6, myogenin, myostatin, and parvalbumin* (Table 3). Based on the largest exon, all genes exhibited high base-pair similarity with the target genome position (88.7 to 99.3%). Of the candidate genes investigated, *growth hormone, myogenin, and paravalbumin* were located on linkage groups containing genome-wide significant QTL. *Growth hormone* on linkage group 16 was 5.3 cM from the nearest QTL marker (QTDT) and 30.2 cM from the nearest QTL peak (GRIDQTL). *Myogenin* on linkage group 3 was 9.6 cM from the nearest QTL marker (QTDT) and 18.4 cM from the nearest QTL peak (GRIDQTL). *Paravalbumin* on linkage group 16 was 25 cM from the nearest QTL marker (QTDT) and 7.8 cM from the nearest QTL peak (GRIDQTL).

**Table 3:** Candidate gene positions on the linkage map. An asterix * indicates that the gene was located on a linkage group also containing a genome-wide significant QTL for growth

Gene	Species	Accession #	Type	LG	cM	bp	% match
growth-hormone*	*Pagrus major*	AB904715.1	DNA	16	53.2	147	99.3
*myostatin*	*Pagrus major*	AY965686.1	DNA	6	38.3	403	99.0
*growth-hormone-receptor*	*Epinephelus coioides*	KR269817.1	DNA	9	58.3	769	88.7
*growth-hormone-receptor-type-I*	*Sparus aurata*	AH014067.4	DNA	9	58.3	760	95.4
*growth-hormone-receptor-type-II*	*Sparus aurata*	AH014068.4	DNA	18	50.3	875	94.1
myogenin*	*Sparus aurata*	EF462192.1	DNA	3	9.6	534	96.8
*myogenic-factor-MYOD1*	*Sparus aurata*	AF478568.1	DNA	7	12.6	591	97.0
*myogenic-factor-MYOD2*	*Sparus aurata*	AF478569.1	DNA	14	39	546	96.5
*myogenic-regulatory-factor-4*	*Epinephelus coioides*	KR269828.1	DNA	15	25.2	510	93.8
*myogenic-regulatory-factor-6*	*Sparus aurata*	JN034421.1	mRNA	15	25.2	521	95.6
*insulin-like-growth-factor-I*	*Sparus aurata*	DQ118098.1	DNA	15	25.2	186	99.5
*insulin-like-growth-factor-II*	*Epinephelus coioides*	KR269813.1	DNA	7	23.4	240	96.7
parvalbumin*	*Sparus aurata*	GU060310.1	mRNA	16	15.2	311	97.7

## Discussion

We assembled the first chromosome level linkage map for the Australian snapper *Chrysophrys auratus*. Proof checking the marker order against the snapper *de novo* genome assembly indicated that the linkage groups were of high quality. QTL mapping revealed eight markers on three linkage groups that were significantly associated with growth. Three candidate genes for growth were located on the same linkage groups as these QTL. These genomic resources will be used to inform the selective breeding program in New Zealand and will form the basis of further genomic investigation in snapper.

Linkage maps are essential for genomic and genetic studies, and have been used extensively to derive the order and spatial position of markers (Cnaani *et al.* 2004; Greenwood *et al.* 2011; Boulton *et al.* 2011). Historically, most first generation linkage maps in fish have been constructed with just a handful or a few hundred markers and did not have genome sequences available to evaluate marker order (Castaño-Sánchez *et al.* 2010). However, technologies advances over the last years have facilitated an increase in the number of markers used to construct linkage maps (Castaño-Sánchez *et al.* 2010; Ninwichian *et al.* 2012) and some have also begun to utilize available genome data for checking both the linkage map and/or genome assembly accuracy (Tsai *et al.* 2016; Wang *et al.* 2017). The snapper linkage map is composed of ∼11K markers and covers all 24 chromosomes of the 738 Mb genome (Figure 2), and is with this marker number the densest to date for the family Sparidae. Even denser linkage maps are being constructed for a few other fish species, for example the linkage map for the Atlantic salmon (*Salmo salar*) includes ∼96K markers, although it should be noted that the genome size in this salmonid species is also significantly larger (2.97 Gb genome size) (Tsai *et al.* 2016). The average correlation between the largest snapper genome scaffolds (bp) and our linkage map (cM) was 0.74 (illustrated for the four largest scaffolds in Figure 3). Comparatively, the results for the recently constructed Atlantic salmon linkage map were 0.81 for the male map and 0.92 for the female map. While the correlation is reasonably high and indicates a good agreement between the map and assembly overall, it is also apparent that some variation around the exact placement of SNPs is apparent at a fine scale (< 5 cM intervals). In many cases this variation is probably the result of inherent precision limitations in the dataset (sample size + number of recombination events), but some of this variation could also be the result of differential recombination patterns across the genome (as observed in Roesti *et al.* 2013).

Using the newly constructed linkage map and available genome scaffolds, we were able to calculate the sex-specific recombination rates for snapper, which showed that females have a higher recombination rate compared to males (female = 3.28 cM/Mb, male = 1.93 cM/Mb). This reflects observations in other fish species, with females often (but not always, Wang *et al.* 2017) having a higher recombination rates than males (Kucuktas *et al.* 2009; Castaño-Sánchez *et al.* 2010; Tsai *et al.* 2016). Overall, the small sex specific differences in recombination rates in this study are consistent with the ranges found in several other fish species including stickleback (*Gasterosteus aculeatus*, 3.11 cM/Mb, (Roesti *et al.* 2013)), Asian seabass (*Lates calcarifer*, 2.4-2.8 cM/Mb, (Wang *et al.* 2017)), and channel catfish (*Ictalurus punctatus*, 2.6 cM/Mb, (Li *et al.* 2015)). Interestingly, it has long been observed that the heterogametic sex shows typically suppressed recombination (Haldane 1922). For example, in *Drosophila* spp., this reduction is so dramatic that during gametogenesis there are no chiasmata being formed and hence no recombination takes place (Morgan 1914). In mammals, species show reduced recombination frequencies in males, which are the heterogametic sex (*e.g.*, humans). It is not known how sex is being determined in snapper, but if the heterochiasmatic sex commonly has a lower recombination rate, then this may indicate that males are heterogametic in this species. Further work is needed to explore this in more detail.

The target trait for this study was growth (Figure 1), which was measured using peduncle length, fork length, and weight (Table 1). Growth is one of the primary targets for selective breeding programs because it relates directly to production output. In fish, it typically has a moderate degree of heritability, and in the current population was shown to be approximately ∼0.26 and ∼0.11 in year one and year three, respectively (Ashton *et al.*, 2019). Other factors that can affect growth include feed amounts, fish density in tanks, and tank design (size, aeration, water flow). We attempted to control for these factors in the current study by standardizing the conditions between tanks and by standardizing measures from each tank.

Genome-wide significant QTL were found in the first year, but not in the third year, which could indicate that the genetic basis of growth in early life is lost as the fish age (Figure 5, Table 2, Supplementary table 1). However, it seems more likely that the lack of growth QTL in year three is due to the decreased sample size from year one to year three. The lower sample size in year three was the result of natural mortality over the course of the study and decreased our number of phenotyped and genotyped fish by roughly half across two time periods. It is notable that the quantitative trait loci of genome-wide significance were highly shared among the three measures (peduncle length, fork length, and weight), which reflects that these are all measures of the same underlying trait (growth). The effect size averaged around 0.05 for individual QTL, which is similar to growth QTL observed in other species including Atlantic salmon (*Salmo salar*: 0.06 to 0.08, (Besnier *et al.* 2015)), tilapia (*Oreochromis niloticus*: 0.06 to 0.19, (Cnaani *et al.* 2004)), chinook salmon (*Oncorhynchus tshawytscha*: 0.14 to 0.33, (Everett and Seeb 2014)), brill (*Scophthalmus rhombus*: 0.08 to 0.12, (Houston *et al.* 2009)), and catfish (*Ictalurus furcatus*: 0.01 to 0.23, (Hutson *et al.* 2014)). The identification of multiple QTL affecting growth indicates a polygenic basis for growth, something that is commonly reported for complex quantitative traits like growth (Wellenreuther and Hansson 2016).

Determining accuracy of QTL placement is an important step in the QTL mapping process as it provides useful information about where variants responsible for an observed QTL signal (*e.g.*, candidate genes or causative alleles) are likely to be located. If high rates of linkage are present between markers, a confidence interval for the QTL region can be estimated - as seen in R/QTL (Broman *et al.* 2003). However, in the current study, pairwise correlation between markers (linkage) across the linkage map indicated very low linkage between markers over even relatively short distances (< 5 cM) (Figure 4). This is most obvious when comparing the linkage observed within the single largest family to that observed in the entire dataset (Figure 4). However, it is worth noting that on linkage groups with genome-wide significant QTL, there does appear to be a number of markers surrounding each QTL that are responding to the QTL signal. As such, it seems likely that there is some linkage between markers at a fine scale (< 5 cM), but that this may be obscured by the low precision of marker placement on the linkage map. If true, the optimal way to get more precise placement of QTL regions will be improved SNP positioning using either a second improved iteration of the linkage map, the genome assembly, or a combination of both these resources. Until this is done it is likely that causative genetic variations underlying the QTL signals will be within this 5 cM scale.

Previous studies have outlined a range of genes and molecular networks that are thought to be candidates for further investigation in teleost species (De-Santis and Jerry 2007). We located the position of candidate genes surrounding the detected growth QTL (Table 3), however, the large distances between the genes and the QTL peaks indicate that one needs to be cautious about a definitive link between the QTL and candidate genes. Our candidate gene search was possible using the available genome sequence data to link gene positions back to their nearest markers. Central to growth in most species is the somatotropic axis, which consists of the *growth hormone releasing hormone* (GHRH), *growth hormone inhibiting hormone* (GHIH), *growth hormone* (GH), and *insulin-like growth factors* (IGF-1 and –II) (De-Santis and Jerry 2007). Of these, *growth hormone* and *insulin-like growth factor I and II* were able to be mapped to the linkage map in the current study. Growth hormone was located near (within 5.3 cM) a QTL of genome-wide significance. In *Sparus aurata*, a close relative of *C. auratus*, a microsatellite repeat in the promoter region has previously been implicated for differences in growth (Almuly *et al.* 2000). This gene would be a good candidate in *C. auratus* because it is close to a QTL in the current study, and the causative microsatellite has been observed in a range of teleost species. Myogenic regulatory factors (*myogenin, MyoD, myf-5, and myf-6*) are another set of potential candidate genes (De-Santis and Jerry 2007). These regulatory factors have been implicated in growth in terrestrial vertebrates, but not in fish species. In this study, the *myogenin* gene was located on linkage group 3 approximately 9.6 cM from a genome-wide significant QTL. In pigs, a polymorphism in the promoter region of *myogenin* was found to account for up to 5.8% of differences in weight (te Pas *et al.* 1999), but no research has investigated its effect in teleost species. A final candidate gene (*paravalbumin*) was located on the same linkage group as a genome wide significant QTL, but was much further away from a putative growth QTL (25 cM) than the previous two genes. A mutation in the promoter region of this gene was previously found to be involved in weight differences in the finfish species *Lates calcarifer*.

### Future directions

While the linkage map constructed in this study can confidently place SNPs in ∼5 cM regions, further work is needed to improve the accuracy of marker placement. More accurate placement of SNPS would help with future work to fine-map and further characterize the QTL and candidate gene locations described in this study. Improved precision should be possible in the near future using the genome assembly that is being further improved by our group. Future work should also aim to detect possible sex-linked markers, to identify regions associated with sex determination, and to investigate sex-specific recombination patterns across the genome. While sex-specific information was not investigated in the current study, this is an area of particular interest in snapper and the data from this study could be used to further investigate it. In conclusion, this study provides valuable genetic and genomic resources for future evolutionary studies and aquaculture breeding programs in this and related species.
